# Expression and Function of S100A8/A9 (Calprotectin) in Human Typhoid Fever and the Murine *Salmonella* Model

**DOI:** 10.1371/journal.pntd.0003663

**Published:** 2015-04-10

**Authors:** Hanna K. De Jong, Ahmed Achouiti, Gavin C. K. W. Koh, Christopher M. Parry, Stephen Baker, Mohammed Abul Faiz, Jaap T. van Dissel, Albert M. Vollaard, Ester M. M. van Leeuwen, Joris J. T. H. Roelofs, Alex F. de Vos, Johannes Roth, Tom van der Poll, Thomas Vogl, Willem Joost Wiersinga

**Affiliations:** 1 Department of Internal Medicine, Division of Infectious Diseases, Center for Infection and Immunity Amsterdam (CINIMA), Center for Experimental Molecular Medicine (CEMM), Academic Medical Center, University of Amsterdam, Amsterdam, the Netherlands; 2 Department of Medicine, Addenbrooke’s Hospital, University of Cambridge, Cambridge, United Kingdom; 3 Mahidol-Oxford Tropical Medicine Research Unit (MORU), Faculty of Tropical Medicine, Mahidol University, Bangkok, Thailand; 4 Center for Tropical Medicine, Nuffield Department of Clinical Medicine, Churchill Hospital, Oxford, United Kingdom; 5 Clinical Sciences, Liverpool School of Tropical Medicine, Liverpool, United Kingdom; 6 Wellcome Trust Major Overseas Programme, Oxford University Clinical Research Unit (OUCRU), Ho Chi Minh City, Vietnam; 7 Chittagong Medical College Hospital, Chittagong, Bangladesh; 8 Department of Infectious diseases, Leiden University Medical Center, Leiden, The Netherlands; 9 Department of Experimental Immunology, Academic Medical Center, Amsterdam, University of Amsterdam, Amsterdam, the Netherlands,; 10 Department of Pathology, Academic Medical Center, Amsterdam, University of Amsterdam, Amsterdam, the Netherlands,; 11 Institute of Immunology, University of Muenster, Muenster, Germany; Massachusetts General Hospital, UNITED STATES

## Abstract

**Background:**

Typhoid fever, caused by the Gram-negative bacterium *Salmonella enterica* serovar Typhi, is a major cause of community-acquired bacteremia and death worldwide. S100A8 (MRP8) and S100A9 (MRP14) form bioactive antimicrobial heterodimers (calprotectin) that can activate Toll-like receptor 4, promoting lethal, endotoxin-induced shock and multi-organ failure. We aimed to characterize the expression and function of S100A8/A9 in patients with typhoid fever and in a murine invasive *Salmonella* model.

**Methods and principal findings:**

S100A8/A9 protein levels were determined in acute phase plasma or feces from 28 Bangladeshi patients, and convalescent phase plasma from 60 Indonesian patients with blood culture or PCR-confirmed typhoid fever, and compared to 98 healthy control subjects. To functionally characterize the role of S100A8/A9, we challenged wildtype (WT) and *S100A9^-/-^* mice with *S*. Typhimurium and determined bacterial loads and inflammation 2- and 5- days post infection. We further assessed the antimicrobial function of recombinant S100A8/A9 on *S*. Typhimurium and *S*. Typhi replication in vitro. Typhoid fever patients demonstrated a marked increase of S100A8/A9 in acute phase plasma and feces and this increases correlated with duration of fever prior to admission. S100A8/A9 directly inhibited the growth of *S*. Typhimurium and *S*. Typhi in vitro in a dose and time dependent fashion. WT mice inoculated with *S*. Typhimurium showed increased levels of S100A8/A9 in both the liver and the systemic compartment but *S100A9^-/-^* mice were indistinguishable from WT mice with respect to bacterial growth, survival, and inflammatory responses, as determined by cytokine release, histopathology and organ injury.

**Conclusion:**

S100A8/A9 is markedly elevated in human typhoid, correlates with duration of fever prior to admission and directly inhibits the growth of *S*. Typhimurium and *S*. Typhi in vitro. Despite elevated levels in the murine invasive *Salmonella* model, S100A8/A9 does not contribute to an effective host response against *S*. Typhimurium in mice.

## Author Summary

Bacterial pathogens are recognized by the host upon infection through interactions between their virulence factors and host cell receptors leading to the activation and recruitment of innate immune cells. *Salmonella* Typhi, the etiologic agent for typhoid fever, however harbors a number of factors, such as a polysaccharide capsule, which prevent the detection of these virulence factors, and thereby dampens the innate host response. Besides bacterial virulence factors, the host can detect endogenous danger molecules which are released upon tissue damage. S100A8/A9, an extracellular protein complex, is such a danger signal that is able to further amplify the systemic inflammatory response upon infection. In the present study we investigated the role of S100A8/A9 during invasive *Salmonella* infection and observed a marked increase of this protein in patients with typhoid fever, which correlates with disease stage and severity. Furthermore we found that S100A8/A9 directly inhibited the growth of *Salmonella* species in vitro thereby functioning as an antimicrobial. When mice were infected with *Salmonella*, the levels of S100A8/A9 were also elevated but mice lacking this protein did not have an altered host response to infection. The role and importance of the elevated levels of S100A8/A9 in human typhoid fever requires further study.

## Introduction

Typhoid fever constitutes a global public health problem with more than 21 million cases worldwide each year resulting in over 217,000 deaths predominantly in Asia [1–3]. Infection with *Salmonella (S.) enterica* serovar Typhi, which is the Gram-negative intracellular bacterium, results in a bacteremic disease characterized by protracted and high-grade fever. Mortality usually results from intestinal perforation and peritonitis or from severe toxic encephalopathy associated with myocarditis and hemodynamic shock [3,4].

*Salmonella* spp. are recognized by pattern recognition receptors (PRRs) including Toll-like receptors (TLRs) [5] and Nod-like receptors (NLRs) [6,7] via conserved motifs termed pathogen-associated molecular patterns (PAMPs) [3]. Important PAMPs of *Salmonella* spp. are the type 3 secretion system (T3SS)-1 and 2, Vi antigen, flagella, lipopolysaccharide (LPS), bacterial DNA and exotoxin [3,8,9]. The presence of the Vi antigen forming a capsule on the cell surface of *S*. Typhi can mask *Salmonella* specific outer surface PAMPs (LPS and flagella) during specific stages of disease [10,11]. The Vi antigen can thereby prevent PRR recognition leading to a reduced cytokine and neutrophil response that occurs in typhoid [12]. The Vi capsule is notably absent in *S*. Paratyphi A, and most other *Salmonella* serovars. It is likely that there are a number of other of mechanisms for down regulating and dampening the host immune responses during enteric fever. We hypothesize that besides PAMPs the host response during typhoid fever is also driven by danger-associated molecular patterns (DAMPs, also called alarmins) that can contribute to PRR-mediated tissue damage in systemic inflammation [13,14].

The S100 proteins S100A8 (myeloid-related protein, MRP8) and S100A9 (MRP14) are DAMPs, which are cytoplasmic phagocyte-specific proteins that represent danger signals and share some functional features of cytokines [15]. S100A8 and S100A9 are able to form stable heterodimers, also called calprotectin, which can elicit a variety of inflammatory host responses once released [16]. S100A8/A9 is a useful biomarker for disease activity in the management of inflammatory bowel diseases (IBD) such as Crohn's disease. In addition, fecal S100A8/A9 can be used to differentiate IBD from irritable bowel syndrome with a high level of accuracy [17]. Most recently, we showed that S100A8/A9 was also up regulated in patients with sepsis and correlated with severity of disease [18].

Previous reports have demonstrated a dual role for S100A8/A9 in severe infection depending on the pathogen it encounters and the stage of disease. Overwhelming release of S100A8/A9 could be detrimental for the host during sepsis contributing to ongoing inflammation, organ damage and/or exhaustion of the immune system [18]. Endogenous S100A8/A9 has been shown to enhance inflammation via activation of TLR4, by amplifying tumor necrosis factor (TNF)-α release in response to LPS, contributing to endotoxin shock-induced lethality [16,18]. However, during early bacterial containment, S100A8/A9 is believed to be important for the host defense against microorganisms by virtue of its contribution in leukocyte migration [19–21] and its direct antimicrobial effects [22,23]. As has been demonstrated by us [23] and others, by chelating trace elements (zinc, manganese) that are essential for bacterial [22–24] and fungal growth [25], S100A8/A9 is able to prevent bacterial growth by altering its metabolism. In a colitis-induced mouse model of salmonellosis, the ZnuABC zinc transporter conferred a significant advantage to *Salmonella enterica* serovar Typhimurium in colonizing the inflamed gut when S100A8/A9 is overexpressed [26]. A recent report has shown that S100A9 can enhance the bactericidal effects of human neutrophils by improving their phagocytosis capacity towards *Escherichia (E.) coli* [27].

The expression and function of S100A8/A9 in typhoid fever caused by *S*. Typhi has not been studied before. We have determined the levels of S100A8/A9 in patients with acute typhoid fever and during convalescence. We have also measured the levels of S100A8/A9 in a murine *Salmonella* model, using wild type and *S100A9* ^*-/-*^ mice, which due to instability of S100A8 in the absence of its binding partner S100A9 are considered deficient for S100A8/A9 at protein level [28,29], and explored the action of recombinant S100A8/A9 on *S*. Typhimurium and *S*. Typhi replication in vitro.

## Methods

### Ethics Statement

The Indonesian patient study protocol was approved by the Indonesian National Institute of Health Research and Development (Litbangkes) and provincial authorities. The Bangladesh patient study protocol was approved by the National Research Ethics Committee of Bangladesh (BMRC/NREC/2010-2013/1543) and the Oxford Tropical Research Ethics committee (OXTREC reference 25–11). Patients and parents of all children recruited to the study gave witnessed, informed and written consent before study enrolment. Animal procedures were performed in accordance with the Dutch Experiment on Animals Act and approved by the Animal Care and Use Committee of the University of Amsterdam (DIX102331).

### Patients

Febrile adults (n = 144) admitted to Chittagong Medical College Hospital, Chittagong, Bangladesh during 2012 were investigated by blood culture, blood real-time PCR for *S*. Typhi and *S*. Paratyphi A and malaria blood smear. There were 28 individuals with blood-culture or PCR confirmed typhoid fever caused by *S*. Typhi. There were no cases with *S*. Paratyphi A detected. Healthy local controls (n = 38) were recruited during the same period. The controls had no documented fever or signs of illness at the time the blood was drawn. In these patients complicated typhoid fever was defined as the presence of one or more of the following: gastrointestinal bleeding (e.g. visible blood in stool); intestinal perforation; encephalopathy (delirium, obtundation, coma); hemodynamic shock; renal impairment (creatinine >2.0 mg/dL); hepatitis (defined as AST or ALT ≥1000 U/L); pneumonia (respiratory symptoms with abnormal chest X-ray) or pleural effusion; presence of focal septic complications; severe anemia (hematocrit ≤20%); need for blood transfusion or in-hospital death [4,30,31]. In Indonesia, 60 patients with blood-culture confirmed typhoid fever caused by *S*. Typhi and 60 healthy local controls were included during a community-based case-control study in Jatinegara, the eastern district of Jakarta, from 2001–2003 [32].

Blood samples were obtained at the time of admission, and day 10 post fever onset or at hospital discharge (Bangladesh study) and at household visits 3 weeks post diagnosis (Indonesian study). Plasma was prepared from EDTA or heparin tubes (BD vacutainer) and stored immediately at −20°C pending analysis. For hospitalized patients, stool samples were taken within 48 hours after hospital admission, and stored at −70°C.

### Experimental Infection and Design

Pathogen-free C57Bl/6 male wildtype (WT) mice were purchased from Charles River Laboratories. *S100A9* ^*-/-*^ mice, backcrossed >10 times to a C57BL/6 background, were generated as described [28], and bred in the animal facility of the host institution. On protein level, intracellular S100A8 is present in *S100A9* ^*-/-*^ mice, however due to a lack of S100A9 as a binding partner these mice do not release S100A8/A9 complexes extracellularly and are therefore considered functional knockout (KO) for S100A8/A9 [28,29]. Age- and sex-matched animals were used in all experiments. For inoculum preparation, *S*. Typhimurium strain 14028 (ATCC) was grown to mid-logarithmic phase in Lysogeny Broth (LB). Mice were starved for 12 hours followed by oral inoculation with 100 μl (10^5^ or 10^6^ colony forming units (CFU)/100 μl HBSS) bacterial suspension as described [33–36]. At 2 and 5 days after infection, mice were anesthetized with Hypnorm (Janssen Pharmaceutical) and midazolam (Roche), blood was drawn into EDTA tubes via cardiac puncture and organs were harvested and homogenized at 4°C. To determine bacterial loads, serial dilutions were plated on horse blood ager (BA) plates and incubated at 37°C for 16 hours.

### Histologic Examination and Immunohistochemical Staining

Liver, spleen, mesenteric lymph nodes (MLN), terminal ileum and cecum were harvested after sacrifice, fixed in 10% formalin and embedded in paraffin. Sections of 4 μm were stained with hematoxylin–eosin (HE), and read by a pathologist who was blinded to groups as described [36]. In short, to score liver inflammation and damage, the entire slide surface was analyzed with respect to the following parameters: area of liver with parenchymal inflammation, necrosis and/or abscess formation, portal inflammation and thrombus formation. Each parameter was graded on a scale of 0 to 4 (0: absent; 1: mild; 2: moderate; 3: severe; 4: very severe). Thrombi were scored as follows: 0: no thrombi; 1: 1–4 thrombi; 2: 5–9 thrombi; 3: 10–15 thrombi; 4: more than 15. The total liver inflammation score was expressed as the sum of the scores for each parameter, the maximum being 16. Spleen sections were scored for inflammation, necrosis/abscess formation, and thrombus formation using the scales given above. Maximum total spleen inflammation score was 12. The cecum, colon, terminal ileum and MLN were assessed for neutrophil infiltration, edema and epithelial disruption to determine whether there was a colitis, adenitis or gut infection present. For S100A8 and S100A9 immunostaining, paraffin sections were dewaxed, blocked with 10% fetal bovine serum, and incubated with rabbit anti-S100A9 antibodies (5 μg/ml) rabbit anti-S100A8 antibodies (5 μg/ml) or rabbit IgG of irrelevant specificity (5 μg/ml, negative control) for 1 hour at room temperature. A Vectastain ABC-AP Kit (Vector) was used for visualization of stained proteins using biotinylated goat anti-rabbit IgG as secondary antibody.

### Cell Culture, Cellular Responsiveness, Phagocytosis and Growth Inhibition Experiments

Peritoneal macrophages (PMs) were harvested as described [37,38]. PMs were seeded on flat-bottomed 96 wells plates at a density of ~50,000/well in RPMI 1640 medium (Gibco) and left to adhere overnight. For whole blood stimulation, 100 μl of heparinized blood was pipetted in a 96 wells U-bottom cell culture plate. Mid-logarithmic phase grown *S*. Typhimurium 14028 was heat-inactivated (HI) at 70°C for 30 minutes. Whole blood and PMs (after multiple washings) were incubated with 1x10^5^, 1x10^6^, or 1x10^7^ HI *S*. Typhimurium or 100 ng/ml LPS (derived from *E*. *coli*; Sigma) diluted in RPMI to a final volume of 200 μl for 4,5 or 20 hours before measurement of cytokine or S100A8/A9 release. Phagocytosis was assayed as described [23,38,39]. In short, HI *S*. Typhimurium was labeled with carboxyfluorescein succinimidyl ester (CFSE, Invitrogen). 50 μl heparinized whole blood was incubated with 50 μl bacteria in PBS (end concentration 1x10^6^ CFU/ml) at 4°C or 37°C. After 0, 15 and 60 minutes, samples were put on ice to stop phagocytosis. Afterwards, red blood cells were lysed using isotonic NH4Cl solution [23]. Neutrophils were labeled using anti-Gr-1-PE (BD Pharmingen) before degree of phagocytosis was determined using FACSCalibur (BD). The phagocytosis index of each sample was calculated as follows: (geometric mean fluorescence × % positive cells at 37°C)—(the geometric mean fluorescence × % positive cells at 4°C) [38].

For the growth inhibition assays, recombinant mouse S100A8/A9 heterodimers were generated as described [40]. *S*. Typhimurium (14028) and the TY21a vaccine strain of *S*. Typhi (Vivotif, Crucell) were grown to log phase and diluted to approximately 10,000 CFU/ml in RPMI 1640 medium. 100 μl of this bacterial suspension was added to 100 μl of recombinant murine S100A8/A9 heterodimer in HBSS (end concentration 50 μg/ml unless indicated otherwise) without Ca^2+^ and Mg^2+^ [23]. Bacteria and S100A8/A9 were incubated for 0, 8, or 24 hours at 37°C. Growth was assessed by plating out ten-fold dilutions of bacterial concentrations on BA plates and overnight incubation at 37°C.

### Assays

Human plasma S100A8/A9 was measured by in-house ELISA and confirmed with Western blots as described [16]. S100A8/A9 was measured in selected fecal samples by ELISA (Bühlmann laboratories). Murine S100A8/A9 complexes (as described previously [23]) and liver TNF-α, interleukin (IL)-6, IL-10 (R&D systems) were measured using ELISAs. Plasma TNF-α, interferon (IFN)-γ, monocyte chemoattractive protein (MCP)-1, IL-6, IL-10 and IL-12 were measured by cytometric bead array (BD Biosciences). Aspartate transaminase (AST), alanine transaminase (ALT), and lactate dehydrogenase (LDH) were measured in plasma with spectrophotometry (Roche Diagnostics).

### Statistical Analysis

Differences between human and controls or murine groups were analyzed by the Mann-Whitney *U* or unpaired *t* test where appropriate. We log-transformed plasma and feces S100A8/A9 concentrations to correct for heteroscedasticity. For linear regression analysis, P value and Spearman rho are reported. Analyses were performed in GraphPad Prism version 6.0 for Mac OS X (GraphPad Software) and data are expressed as mean and SD unless stated otherwise.

## Results

### S100A8/A9 levels in plasma and stool of patients with typhoid fever

**Fig 1:**
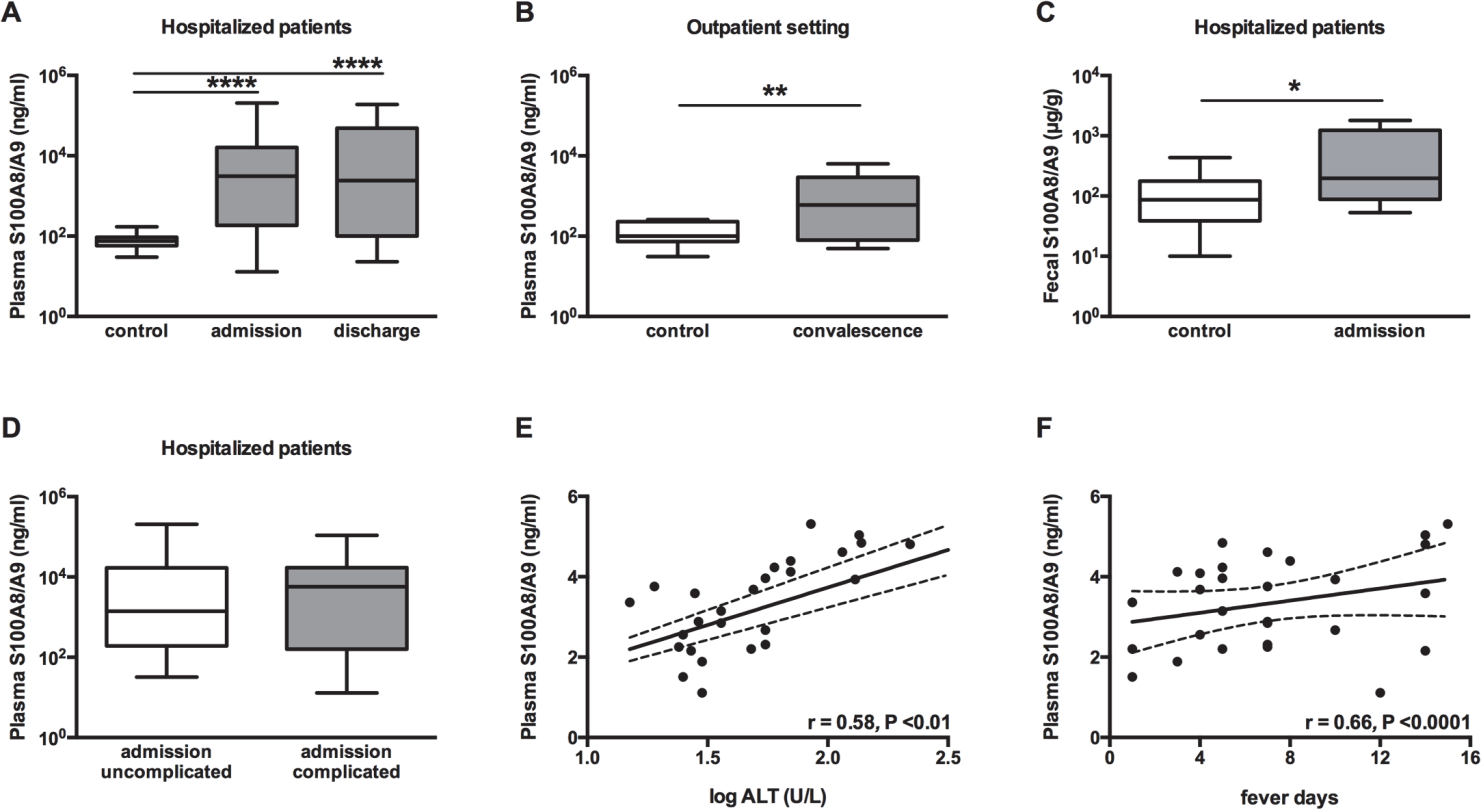
S100A8/A9 complexes are abundantly present in plasma and feces from patients with typhoid fever and correlate with duration of fever. Increased levels of S100A8/A9 (ng/ml) complexes are measured in plasma of patients with confirmed typhoid fever caused by *S*. Typhi admitted to the hospital (A; n = 28 for admission) and compared to healthy controls (n = 38). However S100A8/A9 levels were not yet normalized at time of discharge (n = 15). In a community based control study during convalescence (3 weeks post infection) S100A8/A9 remains elevated (B; n = 60) compared to healthy controls (n = 60). Increased levels of S100A8/A9 (μg/g) measured in feces from hospitalized patients (C; n = 14) compared to healthy controls (n = 36). No differences were seen in levels of S100A8/A9 between hospitalized patients with complicated or uncomplicated typhoid fever as detailed in method section (D). Data are expressed as box-and-whisker diagrams depicting the smallest observation, lower quartile, median, upper quartile and largest observation. Correlation curve log alanine aminotransferase (ALT) versus log plasma S100A8/A9 (E). Correlation curve days of fever prior to admission versus log plasma S100A8/A9 (F). For linear regression analysis, P value and Spearman rho are reported.

We studied 28 hospitalized patients with culture or PCR confirmed *S*. Typhi in Bangladesh and 60 out-patients with culture confirmed *S*. Typhi in Indonesia. The baseline characteristics of both patient cohorts are shown in Table 1. 60 (Indonesia) and 38 (Bangladeshi) healthy volunteers served as control cases. The hospitalized patients had markedly elevated S100A8/A9 in plasma compared to healthy controls (2177.7 ± 14 versus 75.0 ± 2 ng/ml, P<0.0001; Fig 1A). High levels were also found in convalescence in the Indonesian patients compared with local controls (516.4 ± 14 versus 111.9 ± 2 ng/ml, P<0.01; Fig 1B). The S100A8/A9 levels were also markedly elevated in feces of the Bangladeshi patients (602.2 ± 682.3 versus 125.1 ± 115.8 ng/ml, P<0.05; Fig 1C) and the fecal S100A8/A9 correlated with plasma S100A8/A9 levels (r = 0.38, P<0.01).

**Table 1:** Patient characteristics.

	Typhoid fever—acute phase (Bangladesh study)		Typhoid fever—convalescence phase (Indonesian study)	
	Controls (n = 38)	Patients (n = 28)	Controls (n = 60)	Patients (n = 60)
Mean age, years (range)	33.5 (19−55)	28.0 (20−45)	33.5 (4−73)	22.0 (3−57)
Male sex, no. (%)	19 (50.0)	16 (57.1)	24 (40.0)	27 (45.0)
Days of fever (range)	0 (0−1)	7 (1−15)	0 (0)	3 (0−30)
Complications, no. (%)	N/A	11 (39.3)	N/A	0 (0)
Death, no. (%)	N/A	1 (3.7)	N/A	0 (0)

Baseline characteristics of patients with acute typhoid fever (admitted to the hospital, Bangladesh study) or during convalescence (treated in an outpatient setting, Indonesian study). Days of fever prior to admission or when sample was taken, median. Specific complications were: pneumonia (n = 3); encephalopathy (n = 2); gastrointestinal bleeding (n = 1); severe anemia requiring blood transfusion (n = 1); acute hepatitis (n = 1); hepatic abscess (n = 1); septic arthritis (n = 1); and renal failure, cardiopulmonary arrest and death (n = 1). N/A: not applicable.

There were no differences in S100A8/A9 complex levels between uncomplicated and complicated typhoid fever patients (Fig 1D). However, there was a significant correlation between plasma S100A8/A9 levels and liver damage as illustrated by elevated ALT levels (r = 0.58, P<0.01; Fig 1E). In addition, a significant correlation between plasma S100A8/A9 levels and duration of fever prior to admission was observed (r = 0.66, P<0.0001; Fig 1F). S100A8/A9 levels were not yet normalized at time of discharge when patients were afebrile (2630.3 ± 20.0 ng/ml, P = 0.83; Fig 1A).

### S100A8/A9 expression during experimental murine *Salmonella* infection

**Fig 2:**
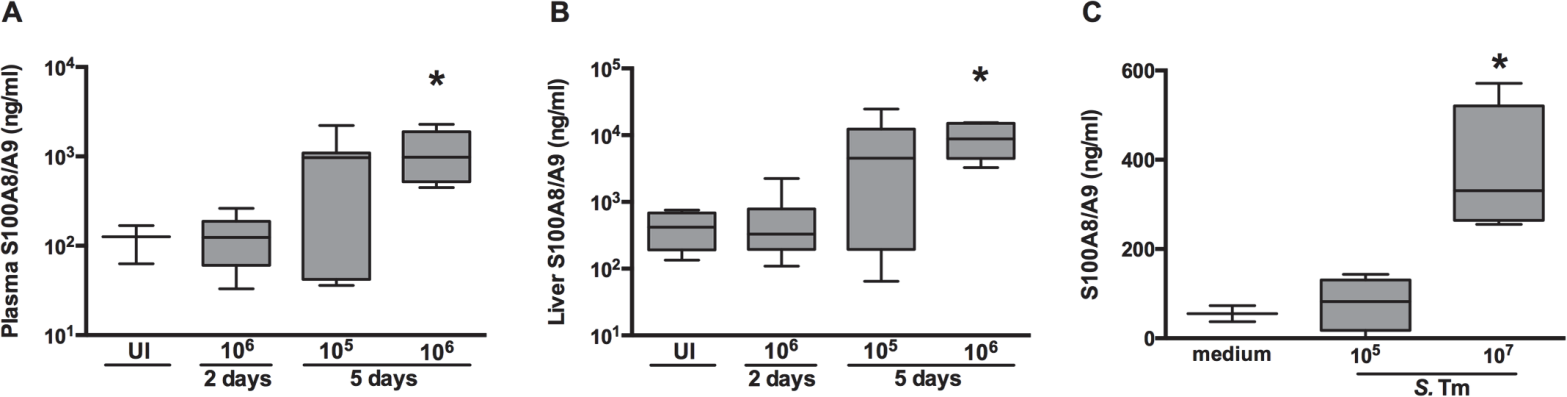
S100A/A9 is up regulated in a murine *Salmonella* model. Levels of S100A8/A9 complexes measured in plasma (A) and liver homogenates (B) from wildtype (WT) mice orally challenged with 1x10^5^ or 1x10^6^ CFU *S*. Typhimurium after 0 (uninfected; UI; n = 3 per group), 2 or 5 days of infection (n = 5–8 per group). (C) S100A8/A9 complexes measured in murine whole blood 4 hours after stimulation with 1x10^5^ or 1x10^7^ heat inactivated *S*. Typhimurium (ATCC) diluted in RPMI to a final volume of 200 μl (n = 4). Data are expressed as box-and-whisker diagrams depicting the smallest observation, lower quartile, median, upper quartile and largest observation. * P<0.05, determined using Kruskal-Wallis testing.

In agreement with the data obtained in patients with typhoid fever, WT mice infected with different dosages of *S*. Typhimurium (10^5^ and 10^6^ CFU) showed increased levels of S100A8/A9 in different compartments corresponding with stage and severity of disease (Fig 2A and 2B). In plasma, low S100A8/A9 levels in uninfected mice (median 126 ng/ml) increased approximately 8-fold 5 days post-infection with *S*. Typhimurium irrespective of the inoculum (median 969 ng/ml with 10^5^ CFU; 980 ng/ml with 10^6^ CFU, P = 0.024; Fig 2A). In liver homogenates, S100A8/A9 levels also increased markedly during the course of infection reaching peak values at 5 days post-infection (4535 ng/ml with 10^5^ CFU and 8827 ng/ml with 10^6^ CFU, P = 0.018; Fig 2B).

**Fig 3:**
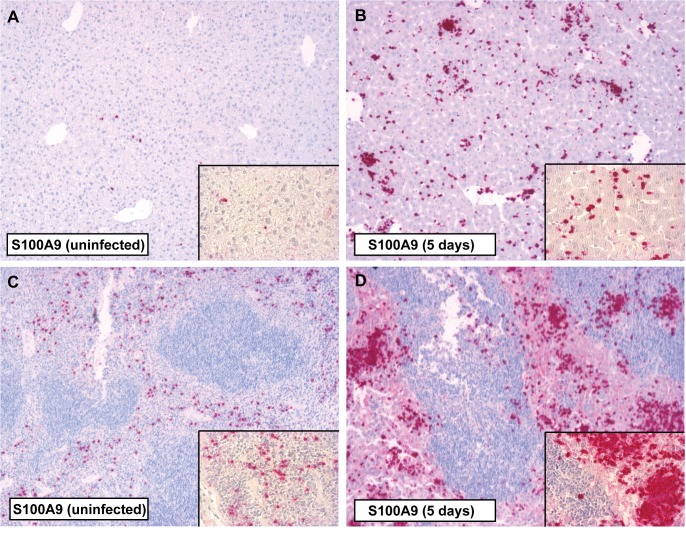
Immunostaining for S100A9 in the liver and spleen of mice during *S*. Typhimurium infection. Positive immunostaining for S100A9 in liver parenchyma (A) and the red pulp of the spleen (C) tissue was observed in uninfected control animals. Five days after infection with *S*. Typhimurium (10^6^) there was a marked increase of S100A9 in both liver (B) and spleen (D) corresponding with inflammatory cell influx. Magnification 10 × and 40 ×.

We next investigated the capacity of murine whole blood cells to release S100A8/A9 in the systemic compartment following infection and found that whole blood from WT mice stimulated for 4 hours with *S*. Typhimurium released S100A8/A9 complexes in a dose dependent manner (P = 0.016; Fig 2C). To further identify the distribution of S100A8/A9 expression during murine *Salmonella*, we performed immunostainings of liver, spleen, and terminal ileum (*Salmonella* entry site). In uninfected WT mice positive immunostaining for S100A8 (S1 Fig) and S100A9 (Fig 3) was observed in liver parenchyma and the red pulp of spleen. After infection with *S*. Typhimurium a marked increase of S100A8 and S100A9 expression was observed in liver (S1 Fig and Fig 3B) and spleen (S1 Fig and Fig 3D) but not at the local site of infection (colon; S2 Fig). Increased S100A8 and S100A9 corresponded with increased inflammatory cell influx (S3 Fig).

### Effect of S100A8/A9 on the cellular responsiveness in vitro

We determined the role of endogenous S100A8/A9 in the inflammatory response to *Salmonella* by analysis of cytokine levels after incubating whole blood and PMs from WT and *S100A9* ^*-/-*^ mice with *S*. Typhimurium for 5 or 20 hours. Concerning the TNF-α response, whole blood derived from *S100A9* ^*-/-*^ mice showed a modest increase after 20 hours compared to controls (P = 0.03; S4 Fig). A similar effect was observed for *S100A9* ^*-/-*^ PMs although this did not reach statistical significance (S4 Fig).

### Effect of S100A9 deficiency on the host response during murine *Salmonella* infection

**Fig 4:**
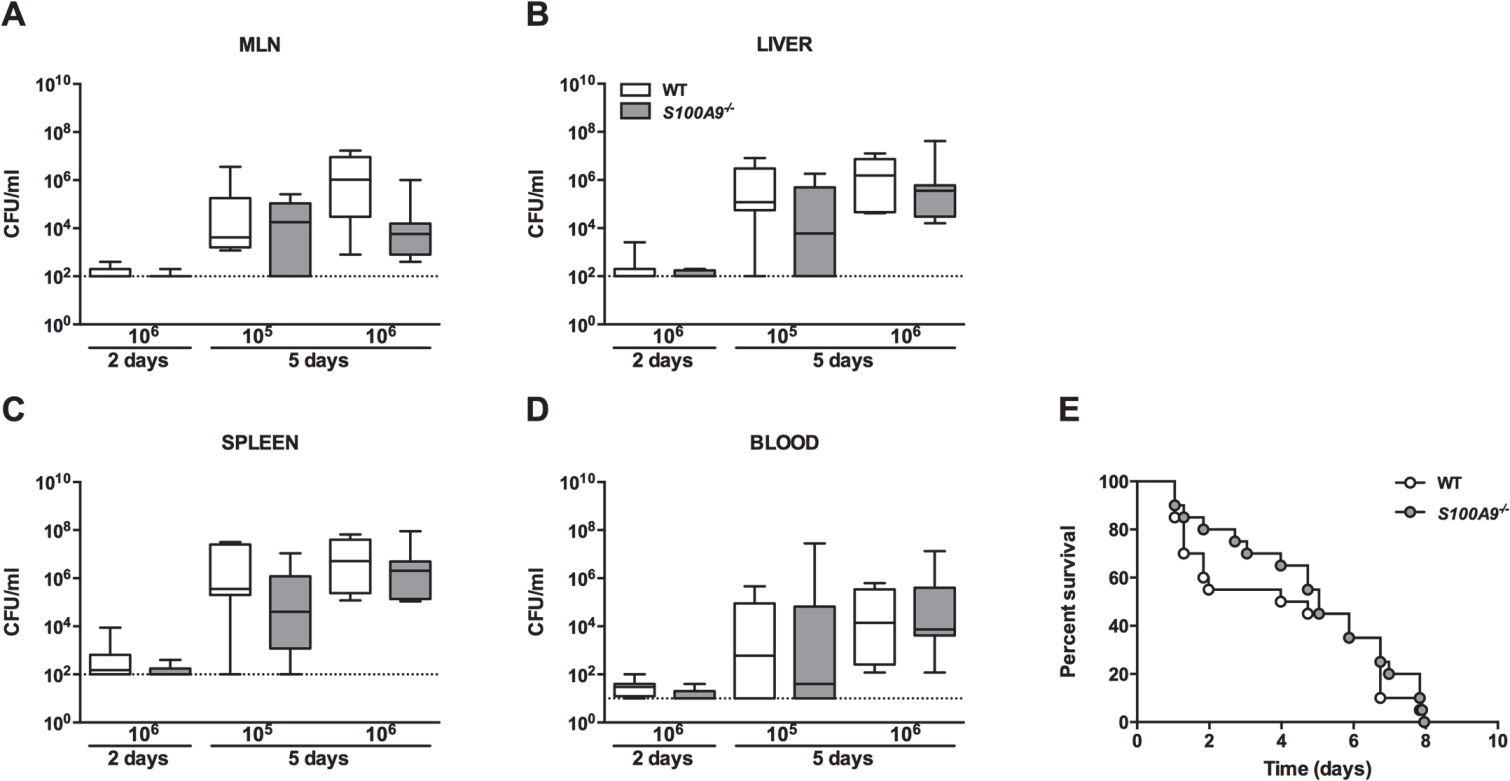
*S100A9* ^*-/-*^ mice are equally susceptible to *S*. Typhimurium infection when compared to wildtype mice in a *Salmonella* mouse model. Bacterial loads in wildtype (WT; white bars) mice compared with *S100A9* ^*-/-*^ mice (grey bars; data represent 5–8 mice per group) from mesenterial lymph nodes (MLN; A), liver and spleen homogenates (B, C), and blood (D), 2 and 5 days post infection with oral 1x10^5^ or 1x10^6^ CFU of *S*. Typhimurium. Data are expressed as box-and-whisker diagrams (log scale) depicting the smallest observation, lower quartile, median, upper quartile and largest observation. Survival curve of WT and *S100A9* ^*-/-*^ mice after oral inoculation with 1x10^6^ CFU of *S*. Typhimurium (E; n = 20 mice per group in each experiment).

We infected WT and *S100A9* ^*-/-*^ mice with 10^6^ CFU viable *S*. Typhimurium and harvested MLN, blood, spleen and liver at predefined time points for quantitative cultures, seeking to collect data representative of local tissue defense, at the primary site of infection, and subsequent dissemination. No differences were observed in bacterial loads at the local site of infection (MLN), nor at distant organ sites (liver, spleen), nor at the systemic compartment (blood), between WT and *S100A9* ^*-/-*^ mice (Fig 4). To investigate the impact of S100A8/A9 complexes on overall survival, we performed an observational study instilling the same infectious dose (10^6^ CFU). This inoculum rapidly led to death within a week for virtually all mice; however, again, no differences were seen between groups (Fig 4E). Arguing that the infectious challenge might have been too high to reveal an effect of S100A9 deficiency during the late time point, we repeated this time point with a 10-fold lower inoculum (10^5^ CFU). However, also with this inoculum size no effect of S100A9 deficiency was seen on bacterial clearance: equal bacterial counts were observed between WT and S100A9 deficient mice in all organs (Fig 4).

### Influence of S100A8/A9 on inflammatory response and organ injury

**Fig 5:**
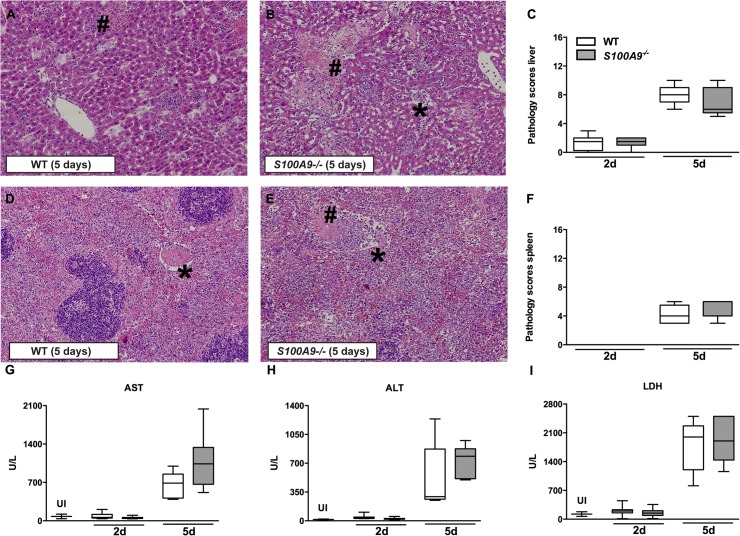
No effect of S100A8/A9 deficiency on organ injury during *S*. Typhimurium infection. Representative slides of liver (A, B) and spleen (D, E) hematoxylin and-eosin (HE) staining of wildtype (WT) and *S100A9* ^*-/-*^ mice, 5 days post infection. Liver and spleen from both WT and *S100A9* ^*-/-*^ mice displayed advanced inflammation with necrosis (#) and thrombosis (*). Magnification 10 ×. Total pathology score was determined at indicated time points in WT (white bars) and *S100A9* ^*-/-*^ (grey bars) mice according to the scoring system described in the methods section (C, F). Aspartate aminotransferase (AST; G), alanine aminotransferase (ALT; H), and lactate dehydrogenase (LDH; I) were measured in plasma. Data are expressed as box-and-whisker diagrams depicting the smallest observation, lower quartile, median, upper quartile and largest observation (5–8 mice per group at each time point).

To study the impact of S100A9 deficiency on cytokine and chemokine release in murine *Salmonella*, we measured cytokine levels (TNF-α, IFN-γ, IL-6, IL-10, IL-12p70) and chemokines (MCP-1/CCL-2) in liver homogenates and plasma harvested from *S100A9* ^*-/-*^ and WT mice after oral infection. In contrast to data derived from a murine endotoxemia model using the same mouse strain [16], but in line with our in vitro data, S100A9 deficiency did not have a marked effect on cytokine and chemokine levels during *S*. Typhimurium infection (Table 2). In liver homogenates, *S100A9* ^*-/-*^ mice displayed a trend towards reduced TNF-α levels after 2 days only; at other time points and for other mediators, levels were similar between groups. Similarly, plasma cytokine levels did not differ between WT and *S100A9* ^*-/-*^ mice at 2 or 5 days post infection with the exception of MCP-1 which was reduced in *S100A9* ^*-/-*^ (P = 0.02) mice 5 days after infection (Table 2). We performed histopathology analyses of liver, spleen and gut in WT and *S100A9* ^*-/-*^ mice infected with oral 10^6^ CFU *S*. Typhimurium. All mice showed evidence of inflammation characterized by diffuse infiltrates, necrosis and thrombosis in their livers (Fig 5A and 5B) and spleens (Fig 5D and 5E); however no differences between WT and *S100A9* ^*-/-*^ mice on total pathology score was seen (Fig 5C–5F). No difference in cecum, colon, and terminal ileum pathology was observed between groups.

**Table 2:** Systemic cytokine profile during experimental *S*. Typhimurium infection in wildtype and *S100A9* ^*-/-*^ mice.

	2 days (10^5^)		5 days (10^5^)		5 days (10^6^)	
	WT	***S100A9*** ^*-/-*^	WT	***S100A9*** ^*-/-*^	WT	***S100A9*** ^*-/-*^
Plasma						
TNF-α	7.2 ± 2.5	21.4 ± 8.3	574.1 ± 212	319.2 ± 148	658.4 ± 177	544.9 ± 160
IFN-γ	15.1 ± 13.5	14.8 ± 4.7	1855 ± 713	634.5 ± 285	1841 ± 535	1759 ± 627
IL-6	19.1 ± 14.8	27.8 ± 12.3	623 ± 223	511.7 ± 215	1334 ± 462	1281 ± 361
IL-10	5.6 ± 1.4	100.5 ± 58.8	25 ± 12.9	9.9 ± 3.214	159.7 ± 149	130.3 ± 124
IL-12	11 ± 1.2	286.5± 158	67.7 ± 35.4	17.6 ± 3.81	39.5 ± 21.73	44.5 ± 14.9
MCP-1	11.5± 2.3	32.9 ± 12.3	985.6 ± 367	316.9 ± 140	2365 ± 572	835 ± 154*
Liver						
TNF-α	113.6 ± 21	73.8 ± 11.9	-	-	494.0 ± 58.3	406.6 ± 93.5
IL-6	31.5 ± 8.6	53.4 ± 6.6	-	-	306.2 ± 28.4	287.7 ± 50.9
IL-10	ND	ND	-	-	209.6 ± 39.5	199.1 ± 65.3

Cytokines were measured in plasma and liver tissue 2 and 5 days after oral infection with *S*. Typhimurium. Data are means ± SEM of 5–8 mice per group. *P <0.05 determined via non-parametric *t* test. Abbreviations: TNF-α: tumor necrosis factor-alpha; IFN-γ: interferon-gamma; MCP-1: monocyte chemo attractive protein-1; IL: interleukin.

Systemic markers of organ injury were elevated during *S*. Typhimurium infection; increased hepatocellular injury (as indicated by elevated levels of plasma AST and ALT), and tissue damage (indicated by elevated plasma LDH), were observed 5 days after infection. However, consistent with the pathology data, these effects were not influenced by S100A9 deficiency (Fig 5G–5I). Taken together, these data indicate that endogenous S100A8/A9 has no effect on the systemic inflammatory response during experimental murine *S*. Typhimurium infection.

### Effect of S100A8/A9 on bacterial growth in vitro and phagocytosis

**Fig 6:**
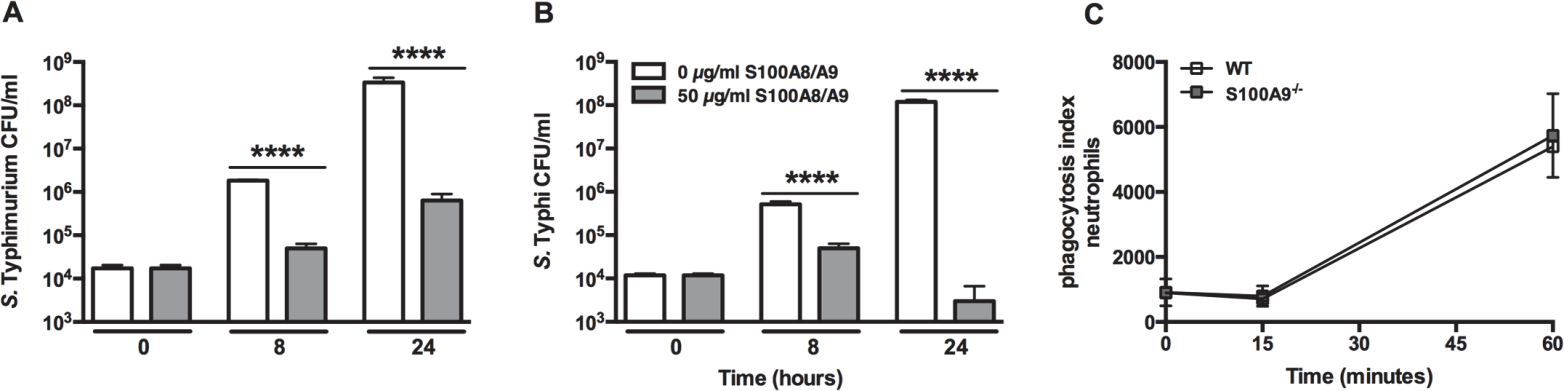
S100A8/A9 inhibits bacterial growth of *S*. Typhimurium and *S*. Typhi in vitro. Growth of *S*. Typhimurium (14028; A) and *S*. Typhi (Vivotif, Crucell; B) was assessed for a maximum of 24 hours in the presence of recombinant S100A8/A9 (50 μg/ml; grey bars) or control (white bars). Bacterial growth was dose dependently inhibited by S100A8/A9. Growth arrested, CFSE-labelled heat-inactivated *S*. Typhimurium bacteria were incubated with peripheral blood neutrophils (C) from wildtype (WT; white) and *S100A9* ^*-/-*^ mice (grey) (n = 4 per mouse strain) for 0, 15 and 60 minutes respectively after which phagocytosis was quantified (see Methods). Data are expressed, as mean ± SD. **** P<0.0001, determined via non-parametric *t* tests comparing different dosages per time point.

We investigated the growth inhibiting properties of S100A8/A9 when encountering either *S*. Typhimurium or *S*. Typhi in vitro. We grew both bacteria in LB medium for up to 24 hours and found that—in line with a previous report [26]—S100A8/A9 disrupts the growth of *S*. Typhimurium in a dose and time dependent manner (P<0.0001; Fig 6A). Similarly, S100A8/A9 was also able to disrupt the growth of *S*. Typhi and even seem to successfully eliminate multiplying bacteria (P<0.0001; Fig 6B). It has been shown recently that S100A9 is able to enhance the bactericidal effects of human neutrophils by improving their phagocytosis capacity [23,27]. We now used neutrophils derived from *S100A9* ^*-/-*^ mice and compared their capacity to internalize CFSE-labeled *S*. Typhimurium with neutrophils derived from WT mice. Although S100A8/A9 is abundantly present in murine neutrophils, in our setting S100A9 did not impact on their capacity to phagocytose *Salmonella* (Fig 6C).

## Discussion

In this study we aimed to characterize the expression and function of S100A8/A9 in typhoid fever, linking observational studies in patients with functional studies in *S100A9* ^-/-^ mice. We here show that S100A8/A9 levels are markedly elevated in Bangladeshi patients with typhoid fever in plasma and feces and correlate with liver damage and duration of fever. The levels were persistently elevated in Indonesian patients 2–3 weeks into convalescence. In a murine *Salmonella* model induced by *S*. Typhimurium increased levels of endogenous S100A8/A9 complexes were seen both at the primary site of infection and at distant sites corresponding with stage and severity of disease. Furthermore S100A8/A9 directly inhibited the growth of *S*. Typhimurium and *S*. Typhi in vitro in a dose and time dependent fashion. The functional role of S100A8/A9 in the host defense against murine *Salmonella* infection was limited however, given the fact that *S100A9* ^-/-^ mice were indistinguishable from WT mice with respect to survival, bacterial organ counts and inflammatory responses.

Typhoid fever is a systemic disease with a disease onset of 7–14 days after oral ingestion with *S*. Typhi [4]. Although macrophages are historically seen as the predominant cells in which *S*. Typhi reside during infection, the bacterium can also be found in a variety of other phagocytic and non-phagocytic cells in vivo, most notably neutrophils [41]. Patients usually present to the hospital toward the end of the first week after the onset of symptoms with fever [4]. A key event in the induction of fever is the release of pyrogenic cytokines (e.g. TNF-α, IL-1β and IL-6) into the bloodstream that mediate the signal leading to a febrile response from the site of inflammation to the thermoregulatory center in the hypothalamus [42]. We and others have shown that these pyrogenic drivers are low during the acute-phase of infection in patients even though high-grade protracted fever is a key feature of typhoid fever [3]. Alternative pathways of the induction of fever have been described [42], with cytokines produced at the tissue level as an alternative pathway for the induction of the signals leading to fever, rather than cytokines in the systemic compartment. Local pro-inflammatory cytokine production in the infected tissue may induce release of secondary mediators with pyrogenic properties. We hypothesize that S100A8/A9 could be such a fever mediator. The very high levels of S100A8/A9 of >200 μg/ml and higher observed in some of our patients are usually only seen in patients suffering from rare syndromes such as Familial Mediterranean Fever [43,44]. Most typhoid fever patients have a normal to low white blood cell count (WBC) suggesting that the observed high concentrations of S100A8/A9 are not due to increased leukocytes counts as observed in other systemic bacterial infections (no correlation between S100A8/A9 and WBC or neutrophil counts was observed). Other potential explanations for the observed high levels of S100A8/A9 in typhoid fever patients could be disturbances in controlled release of these proteins or defective clearance of the proteins [43]. If normal catabolism is assumed the rate of synthesis would need to increase substantially to achieve the measured plasma concentrations, therefore, defective catabolism is a more plausible hypothesis. Catabolism could be reduced because of changes in proteins or cell receptors, but further studies are clearly needed in this specialty. To the best of our knowledge it is currently unknown if one would become febrile upon injection of calprotectin.

The S100A8/A9 complexes are cytoplasmic proteins of phagocytes that elicit a variety of host defense mechanisms. Specifically, S100A8/A9 inhibits the growth of numerous microorganisms by binding of divalent cations [22,23,25]. We previously showed that S100A8/A9 reduces bacterial growth in vitro corresponding to an antibacterial host response against *Klebsiella pneumoniae* in a mouse model of pneumonia-derived sepsis [23]. Similarly, *Salmonella* growth was affected by the presence of this heterodimer in vitro, but in contrast to these earlier studies, S100A9 deficiency did not impact on the systemic host defense in vivo. There are several potential explanations for this discrepancy. One of the functions of S100A8/A9 complexes is to prevent bacterial dissemination in the gut by chelating trace elements. In recent years it has become clear that multiple Gram-negative bacteria such as *E*. *coli* [45] but also *S*. Typhimurium [46] have a ZnuABCzinc transporter and the zinc uptake regulator/repressor Zur. By using a *Salmonella* colitis mouse model others showed that the zinc transporter was required to promote the growth of *S*. Typhimurium over that of competing commensal bacteria indicating that *Salmonella* thrives in the inflamed gut by overcoming the zinc sequestration of S100A8/A9 [26]. Furthermore, S100A8/A9 is known to exert is effects once released extracellular. However since the intracellular *S*. Typhimurium resides mainly within macrophages the role of S100A8/A9 must probably be sought as amplifier of LPS signaling via TLR4. TLR4 plays an important role in the murine *Salmonella* model as TLR4-deficient mice have increased susceptibility to *Salmonella* infection, and stimulation of TLR4 by LPS contributes to the development of septic shock during murine *S*. Typhimurium infection [47]. However, when stimulating whole blood and harvested peritoneal macrophages in vitro with LPS or different dosages of *S*. Typhimurium, only limited differences were seen in the release of TNF-α between WT and *S100A9* ^-/-^. We found numerous circulating S100A8/A9 complexes in mice but no differences were seen in cytokine expression between WT and *S100A9* ^-/-^, which could have pointed towards a role for S100A8/A9 in the LPS-TLR4 mediated signaling during *Salmonella* infection. Finally, circulating S100A8/A9 complexes have been demonstrated to enhance bacterial trapping by neutrophils, however, the formation of these neutrophil extracellular traps (NETs) [25] also occurs at the expense of injury to the host [48]. *S100A9* ^-/-^ mice showed decreased hepatocellular inflammation compared to WT mice in an *E*. *coli* induced sepsis model [18,49]. Bacterial trapping occurs primarily in the capillary beds of the liver and lungs, and excessive NET-formation could lead to hepatotoxicity [50]. In the murine *Salmonella* model we did not find any significant evidence for increased hepatocellular injury in WT mice compared to *S100A9* ^-/-^ mice.

Our study has several limitations. The comparison of acute with convalescence levels in the hospitalized group is limited in that the samples obtained at discharge or day 10 may be too early a timepoint to be a true "convalescent" sample. Therefore, as a comparison, we added the Indonesian study were we look at the convalescence phase in outpatients were blood was drawn during household visits 3 weeks after infection. We have published the details of this Indonesian comparator cohort earlier [32]. Furthermore, the significance of increases in S100A8/A9 is limited by the lack of a non-typhoid febrile control group, which calls into question whether this is a typhoid-specific phenomenon. Another notable limitation is that only serum and fecal assays for S100A8/A9 were performed on typhoid patients, with no cellular responses assayed. Given heterogeneity of S100A8/A9 responses among the patients, this study may have benefited from looking at cellular or cytokine responses to *S*. Typhi antigen stimulation, and determining their associations with S100A8/A9 levels. Some of the complications observed in the typhoid patients in this study, such as hepatic abscess and septic arthritis, are uncommon [4]. Because of the limitations of the facilities available at the study site in Bangladesh it was not feasible to aspirate the hepatic abscess or the septic joint to confirm that *S*. Typhi was the causative pathogen. Based on the clinical picture, we assumed that they are related. The existence of pneumonia due to typhoid is debated and it is possible that pneumonia in our patients was a secondary phenomena caused by another pathogens. Furthermore, care should be taken in translating results obtained from mouse data towards human pathology, especially since *S*. Typhi is an exclusive human pathogen. Although *S*. Typhimurium is widely used in murine studies as a surrogate for *S*. Typhi [3,35,36,51] and levels of S100A8/A9 were both elevated in human typhoid patients and mice infected with *S*. Typhimurium, *Salmonella* spp. have the ability to produce differing clinical manifestations depending on the host infected. For example, increased leukocyte levels and elevated systemic cytokine levels are observed in mice following *S*. Typhimurium infection, but not in typhoid fever patients [8]. It should be emphasized that given the disadvantages of the *Salmonella* mouse model, negative results from the murine model do not exclude a functional role of S100A8/A9 in human typhoid fever.

In conclusion, we here document that patients with *S*. Typhi have markedly increased S100A8/A9 plasma levels which correlate with liver damage and fever duration. Persistent increase in plasma and feces S100A8/A9 levels are observed in acute disease as well as during convalescence. Furthermore S100A8/A9 directly inhibited the growth of *S*. Typhimurium and *S*. Typhi in vitro. In a murine *Salmonella* infection model the levels of S100A8/A9 were also elevated but S100A8/A9 deficiency in mice did not influence bacterial growth and dissemination, organ damage or mortality. The role and importance of the elevated levels of S100A8/A9 in human typhoid fever requires further study.

## Supporting Information

S1 Fig Immunostaining for S100A8 in the liver and spleen of mice. Positive immunostaining for S100A8 in liver parenchyma (A) and the red pulp of the spleen (C) tissue in uninfected control animals. Five days after infection with *S*. Typhimurium (10^6^) a marked increase of S100A8 in both liver (B) and spleen (D) is seen corresponding with increased inflammatory cell influx. Magnification 10 × and 40 ×.
(TIF)

S2 Fig Infection with *S*. Typhimurium did not show a marked increase of S100A8 and S100A9 expression at the local site of infection. Immunostaining for rat IgG (control stain; A), S100A8 (B) and S100A9 (C) of the colon (and feces (*)) in uninfected control animals. Five days after infection with *S*. Typhimurium (10^6^) S100A8 (E) or S100A9 (F) is not markedly increased compared to the control IgG (D) in the intestines. Magnification 10 ×.
(TIF)

S3 Fig Hematoxylin and-eosin and S100A9 staining during murine typhoid fever. Representative slides of liver and spleen for hematoxylin and-eosin (HE; A, C) and S100A9 staining (B, D) in wildtype (WT) mice five days after infection with *S*. Typhimurium (10^6^). Positive S100A9 staining corresponds with HE staining suggesting that the presence of S100A9 increases with inflammatory cell influx. Magnification 4 × and 20 ×.
(TIF)

S4 Fig Effect of S100A8/A9 deficiency on cellular responsiveness to *Salmonella* infection. Tumor necrosis factor (TNF)-α levels after 5 and 20 hour stimulation of peritoneal macrophages (PMs; A, B) and whole blood (C, D) obtained from individual wildtype (WT; white bars) and *S100A9* ^*-/-*^ mice (grey bars) with 1x10^6^ *S*. Typhimurium or *Escherichia (E.) coli* LPS. Data are expressed as box-and-whisker diagrams depicting the smallest observation, lower quartile, median, upper quartile and largest observation (n = 4 per group, per time point). * P<0.05, determined using a non-parametric *t* tests.
(TIFF)
