# Supplementary figures and images for: Expression and Function of S100A8/A9 (Calprotectin) in Human Typhoid Fever and the Murine Salmonella Model
Source: PLoS Negl Trop Dis. 2015 Apr 10;9(4):e0003663. doi: 10.1371/journal.pntd.0003663 (PMC4393299; doi:10.1371/journal.pntd.0003663)

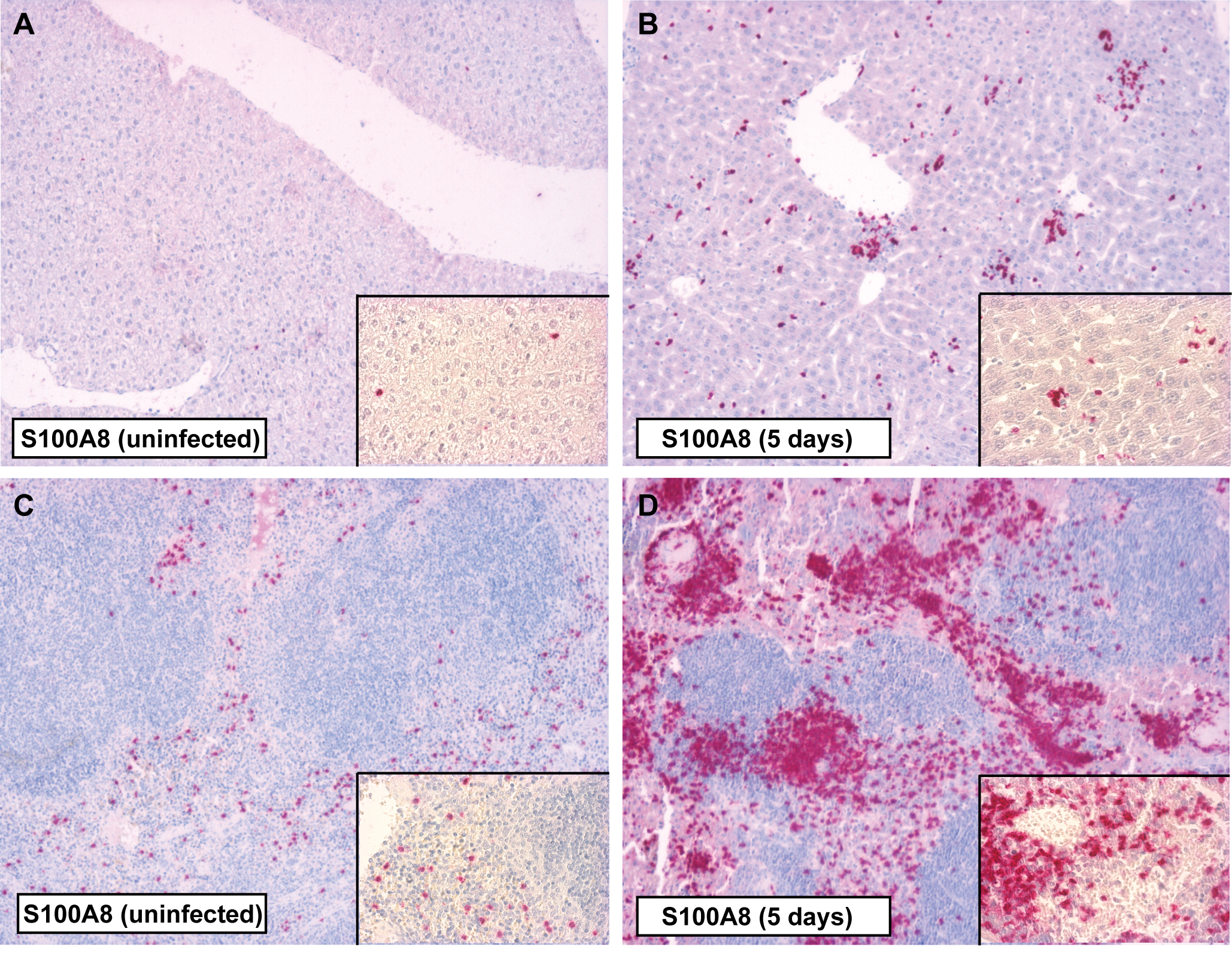

Supplement: S1 Fig — Positive immunostaining for S100A8 in liver parenchyma (A) and the red pulp of the spleen (C) tissue in uninfected control animals. Five days after infection with S. Typhimurium (106) a marked increase of S100A8 in both liver (B) and spleen (D) is seen corresponding with increased inflammatory cell influx. Magnification 10 × and 40 ×. (TIF) [file pntd.0003663.s001.tif]

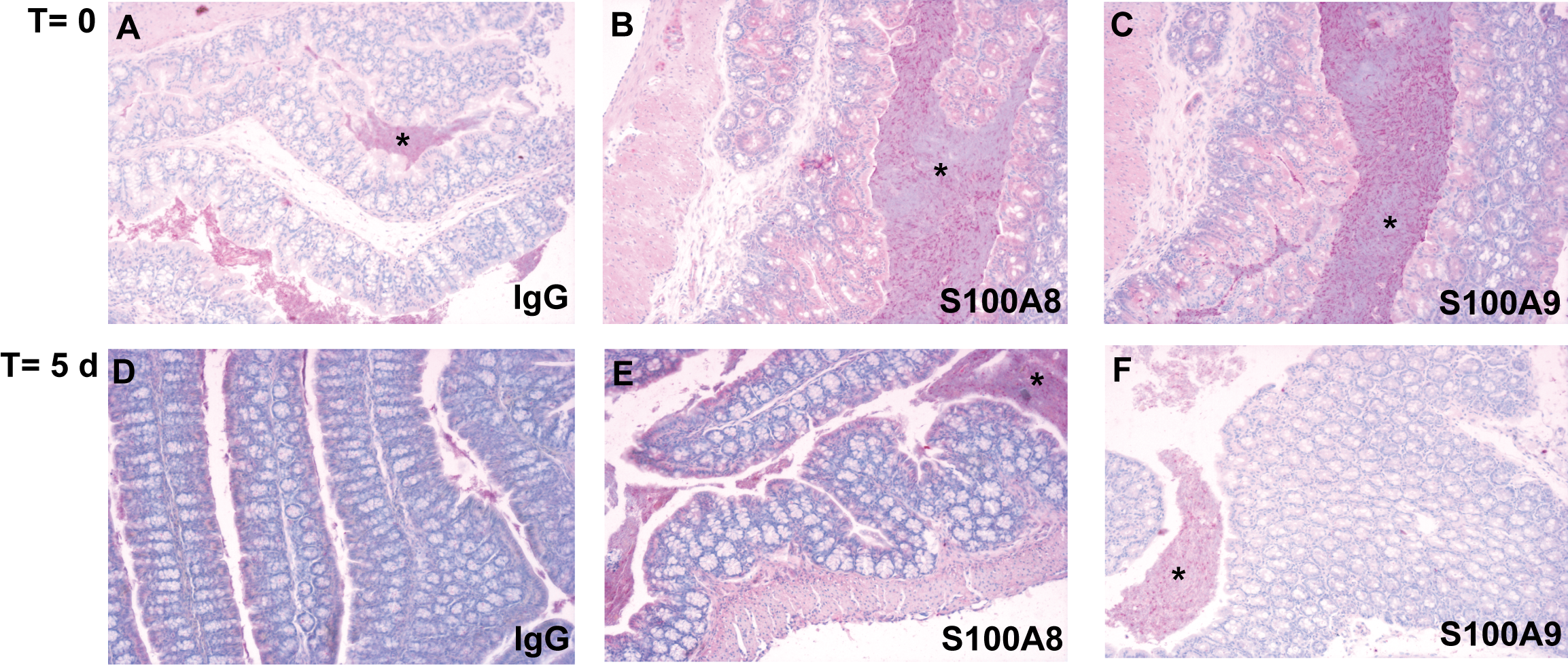

Supplement: S2 Fig — Immunostaining for rat IgG (control stain; A), S100A8 (B) and S100A9 (C) of the colon (and feces (*)) in uninfected control animals. Five days after infection with S. Typhimurium (106) S100A8 (E) or S100A9 (F) is not markedly increased compared to the control IgG (D) in the intestines. Magnification 10 ×. (TIF) [file pntd.0003663.s002.tif]

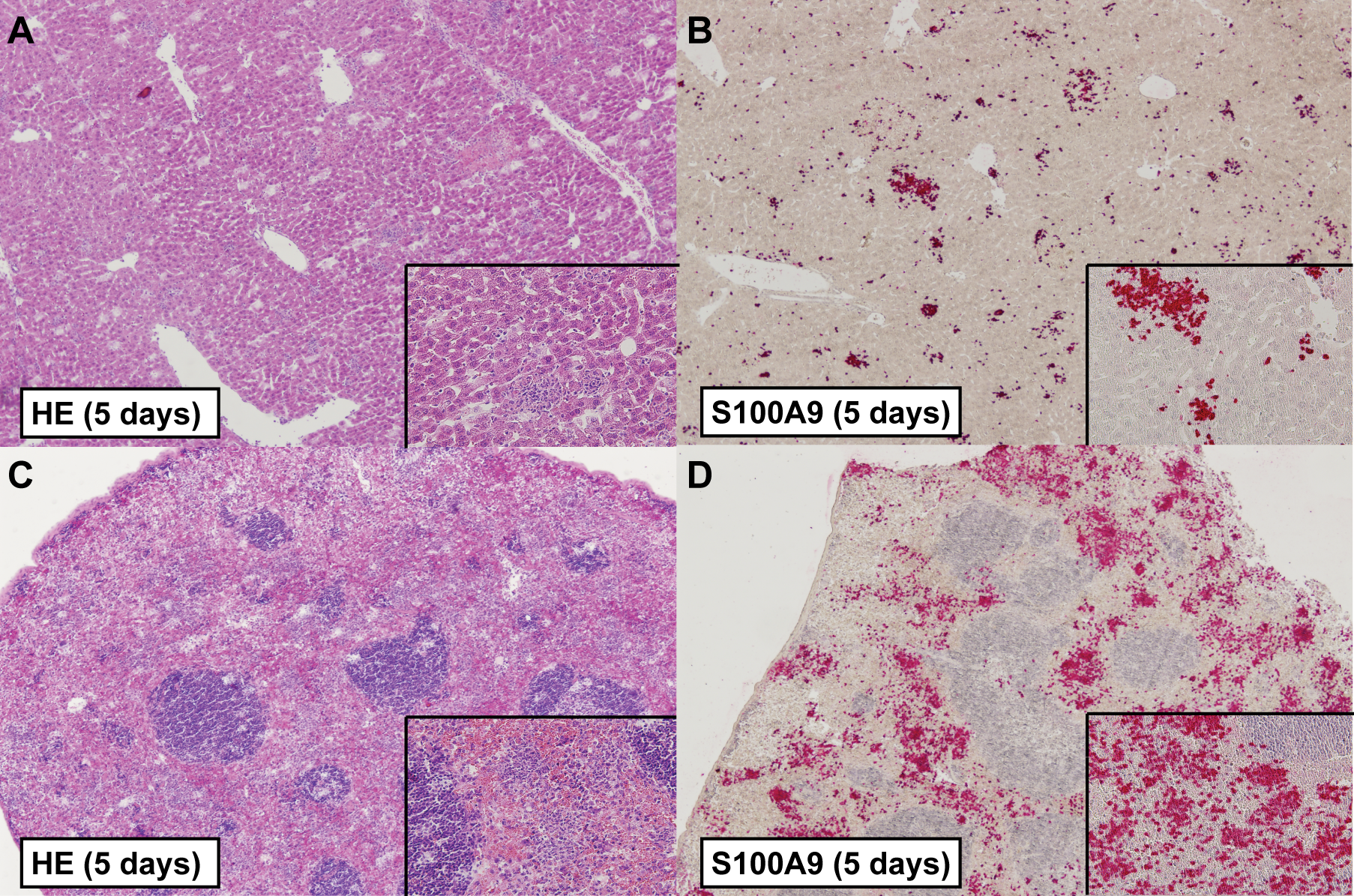

Supplement: S3 Fig — Representative slides of liver and spleen for hematoxylin and-eosin (HE; A, C) and S100A9 staining (B, D) in wildtype (WT) mice five days after infection with S. Typhimurium (106). Positive S100A9 staining corresponds with HE staining suggesting that the presence of S100A9 increases with inflammatory cell influx. Magnification 4 × and 20 ×. (TIF) [file pntd.0003663.s003.tif]

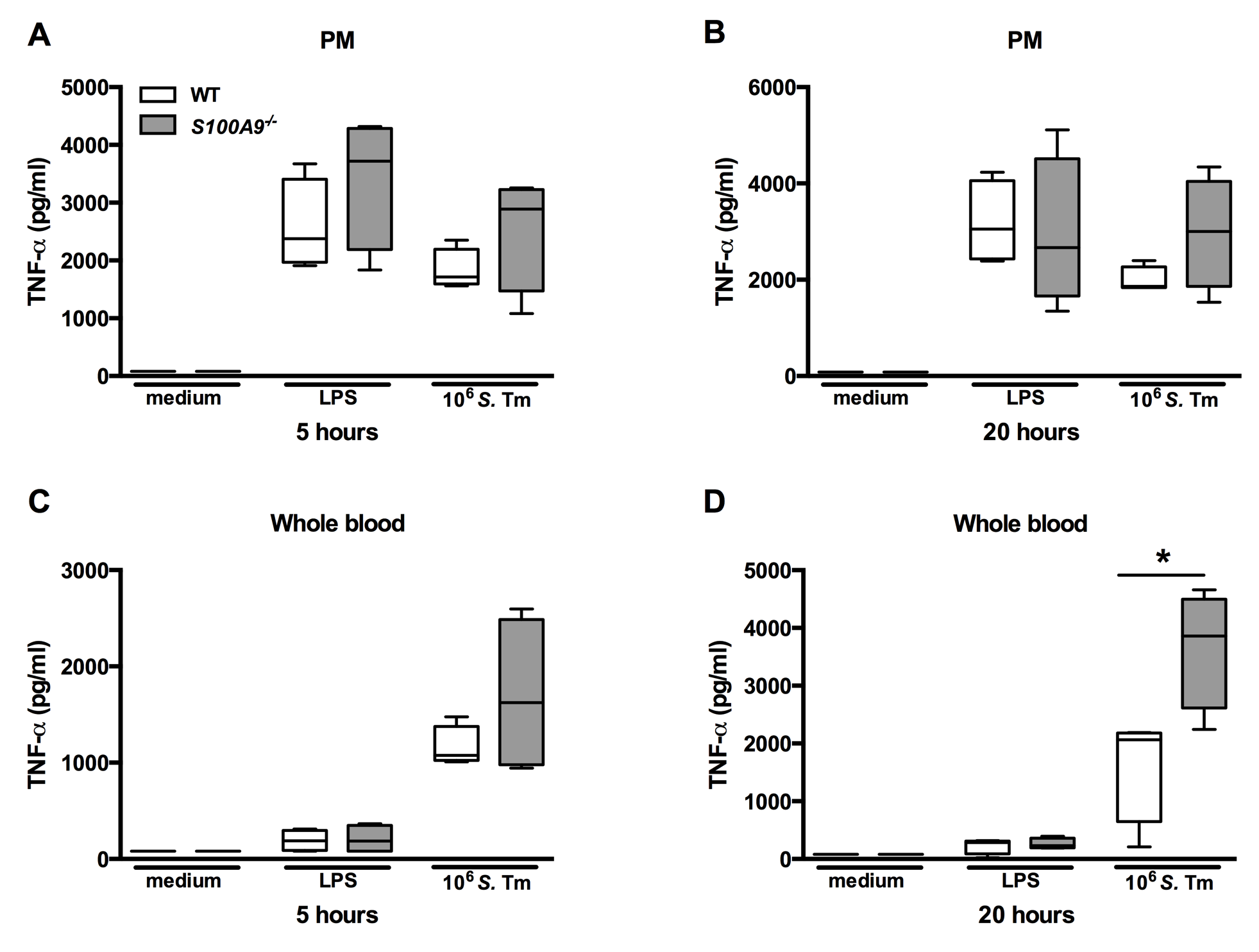

Supplement: S4 Fig — Tumor necrosis factor (TNF)-α levels after 5 and 20 hour stimulation of peritoneal macrophages (PMs; A, B) and whole blood (C, D) obtained from individual wildtype (WT; white bars) and S100A9 -/- mice (grey bars) with 1x10^6^ S. Typhimurium or Escherichia (E.) coli LPS. Data are expressed as box-and-whisker diagrams depicting the smallest observation, lower quartile, median, upper quartile and largest observation (n = 4 per group, per time point). * P<0.05, determined using a non-parametric t tests. (TIFF) [file pntd.0003663.s004.tiff]
